# A Global Perspective on Transition Models for Pediatric to Adult Cystic Fibrosis Care: What Has Been Made So Far?

**DOI:** 10.3390/jcm13237428

**Published:** 2024-12-06

**Authors:** Silvia Cristina Poamaneagra, Doina-Anca Plesca, Elena Tataranu, Otilia Marginean, Alexandru Nemtoi, Catalina Mihai, Georgiana-Emmanuela Gilca-Blanariu, Cristiana-Mihaela Andronic, Liliana Anchidin-Norocel, Smaranda Diaconescu

**Affiliations:** 1 Doctoral School, “George Emil Palade” University of Medicine, Pharmacy, Science and Technology, 540139 Targu Mures, Romania; silviastrat89@yahoo.ro; 2 Faculty of Medicine, “Carol Davila” University of Medicine and Pharmacy, 050474 Bucharest, Romania; doinaplesca@yahoo.com; 3 Department of Pediatrics, Victor Gomoiu Emergency Children’s Hospital, 022102 Bucharest, Romania; 4 Faculty of Medicine and Biological Sciences, “Stefan cel Mare” University of Suceava, 720229 Suceava, Romania; alexandru.nemtoi@usm.ro (A.N.); liliana.norocel@usm.ro (L.A.-N.); 5 “Sf. Ioan cel Nou” Emergency Hospital, 720224 Suceava, Romania; 6 Department of Pediatrics, “Victor Babes” University of Medicine and Pharmacy, 300041 Timisoara, Romania; omarginean@ymail.com; 7 BELIEVE—Center for Disturbances of Growth and Development on Children, 300041 Timisoara, Romania; 8 Department of Pediatrics, Louis Turcanu Emergency Children’s Hospital, 300011 Timisoara, Romania; 9 Faculty of Medicine, “Grigore T. Popa” University of Medicine and Pharmacy, 700115 Iasi, Romania; catalinamihai@yahoo.com (C.M.); georgiana.gilca@gmail.com (G.-E.G.-B.); andronic_mihaela-cristiana@d.umfiasi.ro (C.-M.A.); 10 Department of Gastroenterology and Hepatology, “St. Spiridon” Emergency Hospital, 700111 Iasi, Romania; 11 Faculty of Medicine, “Titu Maiorescu” University of Medicine, 050474 Bucharest, Romania; smaranda.diaconescu@prof.utm.ro

**Keywords:** transition, cystic fibrosis, cystic fibrosis centers, pediatric care, adult care

## Abstract

Interest in the transition of care for cystic fibrosis (CF) patients has grown significantly over time, driven by advancements in treatment that have extended life expectancy. As more CF patients survive into adulthood, the need for structured transition strategies has become a priority for healthcare systems worldwide. Transition programs for CF differ globally, reflecting varying resources and healthcare systems. In North America, the US CF Foundation has fostered adult care since the 1990s, with accreditation standards mandating adult programs and structured transition guidelines, exemplified by the CF RISE program for gradual responsibility shifts. Canada integrates US-inspired models, emphasizing national advocacy and outcomes evaluation. In Europe, approaches varies widely; the UK leads with structured programs like the Liverpool model and robust registry support, while France and Germany adopt multidisciplinary methods. In Australia and New Zealand, youth-centered policies prioritize early planning and access via telemedicine. In Asia, where CF is rare, transitions are less formalized, with some progress in countries like Japan and Turkey, though resource gaps and limited data tracking remain significant challenges. Despite varied approaches across countries, common barriers like resource limitations and psychological readiness continue to challenge successful transitions. Highlighting the importance of centralized, well-coordinated transition programs, recent initiatives have focused on the implementation of national and international CF registries to enhance health outcomes and quality of life. This narrative review provides a global perspective on transition strategies developed across various healthcare systems for CF patients, identifying best practices, common challenges, and outcomes related to the continuity of care.

## 1. Introduction

Cystic fibrosis (CF) is the most common autosomal recessive disease in the Caucasian population, and it is caused by mutations in the CF transmembrane conductance regulator (CFTR) gene [1]. CF occurs in 1:2000–3000 live births in Europe with the total affected population being estimated at 32,000 patients in Europe and 30,000 in the United States [2,3]. The survival age in developed countries ranges from 44 to 53 years, due to new advances in CF care including early diagnosis through newborn screening programs, targeted therapies, airway clearance therapies, introduction of pancreatic enzyme replacement, and nutritional support [4].

The discovery of the CFTR gene four decades ago triggered extensive research leading to the introduction of CFTR modulator therapies [5,6].

In Canada, 60% of CF patients have already reached adulthood, a proportion that has doubled in the last 35 years [7]. Similar data are available from the European CF Society Patient Registry which estimates that between 2010 and 2025, there will be a 75% increase in the CF adult population; however, it seems that these percentages are underestimated [8]. Advancements in understanding and treatment, along with exceptional pediatric care, have transformed CF from a predominantly pediatric condition into an adult disorder, with the majority of mortality now occurring in the adult population [9]. This demographic shift highlights the need for high-quality transition programs that provide integrated healthcare as individuals progress from adolescence to adulthood [10].

CF requires continuous management to prevent complications and optimize long-term outcomes. In pediatric care, treatment often focuses on growth, development, and family-centered care, whereas adult care emphasizes self-management, long-term disease control, and addressing broader life concerns such as employment, relationships, and mental health. A well-structured transition process ensures that patients receive the necessary support to manage their condition independently into adulthood [11].

As life expectancy for CF patients continues to increase, complications such as lung function decline, diabetes, and liver disease become more common in adulthood, requiring specialized adult care. Without proper transition, gaps in care can lead to poor disease management, increased hospitalizations, and reduced quality of life [12]. Care provided in specialized centers has become standard practice in high-income countries, enabling patients to access the expertise of complex care teams [13].

A global examination of CF transition models is essential due to the wide variation in healthcare systems, resources, and practices across different countries. Approaches to transition differ significantly between high-income countries with well-established CF care centers and low- to middle-income countries where CF care infrastructure may be underdeveloped or fragmented [10]. Exploring global transition models allows for the identification of best practices that can be adapted to fit diverse healthcare environments, ensuring that all patients, regardless of where they live, receive appropriate and effective care as they age.

The aim of this narrative review is to assess current transition models for CF care worldwide by examining the approaches, challenges, and outcomes associated with this critical shift. By examining CF transition models on a global scale, healthcare providers and policymakers can collaborate to enhance the quality of care and long-term health outcomes for CF patients transitioning to adulthood, regardless of geographic or economic disparities.

## 2. Components of a Successful Transition in CF Care

On the one hand, the transition process has been defined as “a purposeful, planned movement of chronically ill adolescents and young adults from the family-centered to the adult-oriented healthcare systems” [14]. On the other hand, the transfer represents a discrete event occurring during the transition, involving the bureaucratic shifting from the pediatric to the adult care team [15].

Initially, transition policies were informal or nonexistent, leading to gaps in care and poor health outcomes [16]. However, as the adult CF population increased, so did the recognition of the importance of smooth, well-coordinated transitions. Healthcare systems have adapted to the growing adult CF population in various ways, leading to the development of formal transition programs [17]. These strategies often involve preparing patients from an early age, promoting self-management skills, and ensuring effective communication between pediatric and adult care teams. Health organizations have also emphasized the need for specialized adult CF centers, which can address the unique needs of adult patients, contributing to improved long-term outcomes and quality of life [18]. In some cases, centralized CF care has continued, integrating both pediatric and adult services. However, many countries now favor the establishment of independent adult CF centers, leading to a rapid increase in the number of such specialized facilities [19].

As individuals with CF experience a range of multi-system complications, particularly involving pulmonary and nutritional aspects, the care team has traditionally included a physician (often a pulmonologist), along with a program coordinator, a nurse, a respiratory therapist, a dietitian, and a social worker. In recent years, additional specialists, such as endocrinologists, gastroenterologists, and mental health professionals have become more involved in managing the growing number of CF-related complications [20,21]. These specialized multidisciplinary teams focus on optimizing growth and development, minimizing disease progression, managing CF complications, providing ongoing support, sexual and reproductive education, and integrating specific therapies into the changing everyday life of CF patients [22].

Experts recommend that transition teams adopt a collaborative approach to encourage treatment adherence. This approach should emphasize empathetic communication and provide clear, evidence-based information on the action of therapies and the disease progression in the absence of medication. Adherence to treatment should be addressed during every consultation to reinforce its importance [23,24].

Supporting patients with psychologists and social workers in the transition team is essential. These professionals can identify and address perceptual or emotional barriers that may hinder adherence, particularly in patients who deny conflict situations or lack the motivation to follow a therapeutic plan [25].

The optimal timing for initiating the transition process in CF patients remains a subject of ongoing debate. The age at which transition preparations commence varies across countries and, in some cases, within the same country, influenced by socio-economic disparities and differences in access to healthcare services [10,12,17,25,26]. According to a recent international study, the initiation of the transition process occurs between 16 and 21 years of age, reflecting the diversity in healthcare practices globally [27].

Some authors recommend initiating the transition during early adolescence, around 12–13 years of age, through active information exchange, individual consultations, and promoting self-management skills. The initial phase involves discussing the transition plan with both patients and families, continuously updating it until the transfer of care [10,28].

Another critical aspect is the coordination between pediatric and adult teams, ideally through formal transition clinics where available. These meetings help create individualized, phased transition plans, covering topics such as diagnosis, growth, lung function, psychosocial dynamics, and self-management progress. A comprehensive transition letter prepared by the pediatric team, in collaboration with the multidisciplinary group, is recommended. This document should include details about physiotherapy, diet, medical, and social support programs [29].

Some authors further advocate for a dedicated transition coordinator to serve as the primary contact for the family and patient, offering personalized support at least six months before to six months after the transfer [30]. This individual meets with the patient’s family around the age of 14, fostering trust, guiding the young person through changes in care, ensuring their adaptation to adult life, including education or employment [27]. The coordinator also attends early appointments of adult care, supports the first hospital admission, and helps integrate patients into academic or professional settings [31].

## 3. Regional Approaches to Transition

### 3.1. North America (US and Canada)

In the United States (US), the CF Foundation began actively supporting the development of adult CF programs in the late 1990s. As part of its accreditation process, the CF Foundation mandates that care centers treating more than 40 individuals over the age of 21 must maintain a dedicated adult CF program. CF Foundation-accredited care centers typically consist of both pediatric and adult programs and may also include affiliate programs that predominantly manage pediatric patients, with each affiliate program linked to a main care center for oversight [32,33].

In preparation for the transition to adult care, the CF Foundation provides specific guidelines for the year prior to transfer. These recommendations include at least four clinic visits, four sputum cultures, and two pulmonary function tests, ensuring comprehensive monitoring and readiness for adult care transition [23,34].

The US healthcare system allows for flexibility in choosing healthcare programs; this system enables adult patients to remain in pediatric programs under certain circumstances. Disparities in the availability or proximity of adult care programs, particularly those specializing in CF care, may lead some patients to remain in pediatric settings well into adulthood. This flexibility is further supported by the absence of a strict, national mandate requiring transfer to adult care at a specific age, allowing for decisions based on individual needs and program resources [35,36,37].

A national American study examining CF patients transitioning to adult care between 2007 and 2013 analyzed the following covariates: the patient’s pediatric program’ location compared to the adult care one, the type of program from which the patient transferred (pediatric or affiliate), whether the patient had consulted with a social worker during their final year of pediatric care, and whether an oral glucose tolerance test was performed in the patient’s last year of pediatric care. A total of 3818 patients did not transition to adult care and remained in pediatric programs. Of these, 18% were 17 years old, 55% were between 18 and 25 years old, and 27% were 26 years or older. In the group of patients who transitioned to adult care, the mean age at the time of transfer was 21.1 years, with 68% transferring between the ages of 18 and 21. The average gap in care during the transfer process was 183 days; a total of 13% had a prolonged gap of 365 days or more. Prolonged gaps in care were more frequently observed in patients who transferred before the age of 18 and in those who transitioned to an adult care program located in a different city from their pediatric or affiliate program [32].

The CF RISE program (responsibility, independence, self-Care, and education) is a structured and carefully planned transition initiative designed to enhance quality of life, promote patient independence, and ensure the continuity of care during the transition from pediatric to adult services. Since its implementation in the US in 2015, the program has provided essential tools and resources for both patients and caregivers to foster a comprehensive understanding of CF [10,38]. This program is intended to facilitate a gradual, deliberate transfer of responsibility from caregivers to patients, while supporting clear communication between pediatric and adult care teams, patients, and families [39]. The program encompasses the following six key responsibility areas: collaboration with the CF care team and other healthcare professionals, managing CF treatments, living with CF, navigating the CF transition process, planning for school and career, and understanding finance and insurance matters [40]. Despite the program’s structured approach, there is currently no universally adopted, standardized, evidence-based model for transitioning CF patients from pediatric to adult care [41].

While some Canadian CF clinics are looking to integrate ON TRAC’s (transitioning responsibility to adult care) transition clinical pathway, CF Canada prioritized transition as a national advocacy initiative, leading the project to adapt the CF RISE transition program from the US [42].

At the British Columbia Children’s Hospital, a CF transition protocol was implemented in 2000 and has evolved into a multi-faceted transition program. A 10-year retrospective evaluation of CF patients who transitioned from British Columbia Children’s Hospital to the adult CF clinic revealed strong patient attendance at their designated transition clinics, along with a high completion rate of medical transfer summaries. The evaluation also identified areas for improvement, such as reducing the time gap between the final pediatric appointment and the first adult clinic visit [43,44].

The Canadian Association of Pediatric Health recommends that key population health indicators should be evaluated, including adherence to medication pick-up and disease-specific outcomes, such as FEV1 measurements, tracked in the two years before and after the transition to adult care. Additional metrics involve the development of self-care skills, as reflected by the achievement of transition pathways goals and readiness indicators. Measures of attachment to adult CF clinics, such as the frequency of clinic appointments within the first two years post-transfer, are also important markers of a successful transition [26,45,46].

### 3.2. Europe

The European Reference Network recognized the paramount importance of transitional care and transitional research through the Directive 2011/24/EU of the European Parliament and the European Council [47,48].

The European Respiratory Society (ERS) released an ERS statement in July 2024 on transition of care in childhood interstitial lung diseases outlining key strategies to address the needs of adolescents as they transition to adult care systems. This guidance recommends individualized transition plans tailored to each patient’s medical and psychosocial needs, developed with both pediatric and adult healthcare teams. Emphasizing patient education and empowerment, it encourages patients and families to become knowledgeable about managing their health. These guidelines recommend a collaborative approach, including regular assessments and harmonized care protocols between pediatric and adult systems [49].

Countries with strong CF networks, such as the United Kingdom (UK), France, and Germany, benefit from specialized CF centers with well-established multidisciplinary teams and structured transition programs supporting patients from pediatric to adult care. These countries often have comprehensive national CF registries and standardized care protocols, ensuring high-quality, consistent care across regions [50,51]. In contrast, countries with fewer resources or smaller CF populations may struggle with limited access to specialized care, fewer dedicated centers, and less formalized transition programs [52].

The UK pioneered structured transition programs in Europe, with studies from various countries reporting positive outcomes associated with these programs. The UK has also led globally in establishing adult specialist centers distinct from pediatric care, a model endorsed by the European Respiratory Society in collaboration with the European CF Society through a task force report [53].

The UK CF Registry, a national and secure database sponsored and managed by the CF Trust, was established in 1995. It collects demographic and longitudinal health data on nearly all individuals with CF in the UK, currently encompassing over 12,000 patients, with a coverage rate of more than 99% of the CF population in the country [51,54].

In 1965, the first adult CF clinic in the UK was established at the Royal Brompton Hospital in London. Today, several CF clinics across the UK have integrated both pediatric and adult CF programs, offering comprehensive care that includes lung transplantation services [55].

In the UK, the latest National Institute for Health and Care Excellence (NICE) guidelines on the transition from pediatric to adult care for adolescents with chronic conditions were published in 2016. These guidelines emphasize a person-centered and coordinated approach, tailored to the individual needs of each patient. The transition should be planned early, typically beginning at age 13 or 14, with the actual transfer occurring between 16 and 18 years of age. Key principles include involving the adolescent in decision-making, ensuring a gradual and structured transition process, and providing appropriate information and support for both the young person and their family [56].

One example of a planned transition is the Liverpool model, where the adult care is provided by a regional CF unit, such as the Liverpool Heart and Chest Hospital. This program involves three phases: pre-transition starting at the age of 13, transition starting at the age of 17 and going on until the patient is integrated into adult care, and the post-transition phase which includes annual reviews. In the year preceding the planned transition, members of an interdisciplinary team from both adult and pediatric services convene to review and discuss the management of each transitioning patient. The patients are subsequently contacted and offered an individualized, informal visit to familiarize themselves with the adult care facilities. The formal transfer of care occurs during joint visits held annually at the adult center; each patient attends at least two of these events [57].

In France, multidisciplinary CF centers were created in 2002 and integrated physicians, nurses, physiotherapists, dieticians, psychologists and social workers who specialized in CF care [50,58,59]. In 2022, there were 47 French CF centers associated with a national CF network known as the “filière Muco-CFTR”; these centers were either pediatric, adult, or mixed centers [60,61]. Despite this complex CF care infrastructure, there are no French national guidelines for the transition of CF patients from pediatric to adult care. Several authors investigated the transition process aiming at its improvement [50,61].

The SAFETIM (Suivi de l’Adolescent, de sa Famille et des Equipes vers une Transition Idéale dans la Mucoviscidose) project investigated the transition process in French CF centers [62]. The process began around the age of 15 and lasted between 3 and 5 years. Researchers recommend identifying a common caregiver to accompany the families, scheduling appointments to the adult services from the pediatric clinic, organizing visits to the adults’ department, providing enough preparation time by the early initiation of the process, and using indicators to evaluate the progress [50,63].

The first Italian CF center was established in 1968 at the Gaslini Institute in Genoa, marking the beginning of organized CF care in Italy, with a particular focus on multidisciplinary approaches to improve patient outcomes, which eventually led to the establishment of more specialized CF centers across the country [64]. The Italian CF Foundation (Fondazione Ricerca Fibrosi Cistica) supports such centers to ensure comprehensive, lifelong care, with models that help streamline transitions from pediatric to adult CF care [65].

The Italian Society of Pediatric Gastroenterology, Hepatology, and Nutrition together with the Italian Association of Hospital Gastroenterologists and Endoscopists, the Italian Society of Endoscopy, and the Italian Society of Gastroenterology, convened a panel of experts to create a set of practical recommendations for establishing an effective roadmap for transition practices in gastroenterology. While challenging to determine precisely, the optimal age range for transitioning was recommended to be between 16 and 20 years, depending on factors such as physical and emotional maturity, disease activity, adherence to treatment, and autonomy in disease management, according to the recommendation of category 2B. The same experts recommend that the transition process should include a period of overlap where both pediatric and adult care providers are involved; the duration of this overlap should be adjusted according to the disease severity [66].

In Germany, the quality of care for CF patients has gained significant focus. Prior to the unification of East and West Germany in 1990, CF patients were treated in over 100 non-accredited outpatient clinics and private practices. Patient data were submitted annually to separate registries in East and West Germany. In 1995, the establishment of the CF Quality Assurance project marked a commitment to enhance CF care in Germany by focusing on quality improvements across care structures, processes, and outcomes [67,68].

In a recent study, members of the European Respiratory Society were assessed via an online survey and the authors gathered responses from 44 centers (35 European centers and 9 non-European ones) regardless of their dedication to exclusively care for CF patients. A structured transition program between pediatricians and pulmonologists had been implemented in 69% of the centers. Multi-specialty teams were available at the time of transition in 81.4% of the centers. Regarding patient education, 59.5% of the centers provided written informational materials, such as brochures. All surveyed centers maintained communication channels for patient–physician contact. The most frequently used communication method was a landline phone (80.9%), followed by email (78.6%), and text messaging via phone applications (33.3%). Additionally, 11.9% of the centers reported using mobile applications, social networks, or personal phones for communication [69].

Teams in the Netherlands have successfully implemented transition programs using virtual meetings; the authors recommend this method as it appears to be an effective mode of communication for CF teams [70]. Another Dutch study evaluated a structured transition clinic for CF adolescents moving to adult care. Using a controlled observational approach, researchers compared health outcomes between patients using the transition clinic and a control group. The clinic employed standardized protocols, multidisciplinary support, and education tailored to foster patient independence, including individual consultations with the adult team. The results indicated the transition clinic model significantly improved health markers and patient satisfaction, underscoring the effectiveness of structured transition programs in enhancing health outcomes for CF patients in the Netherlands [71,72].

Establishing national CF registries is highly recommended, especially as they play a critical role during the transition from pediatric to adult CF care. These registries facilitate the systematic tracking of patients’ health over time, allowing healthcare providers to closely monitor changes in treatment effectiveness, disease progression, and complications through different life stages [73]. In Europe, well-developed registries in high-income countries provide comprehensive, regularly updated data, which is essential for improving transition practices and supporting the continuity of care. However, in low- and middle-income countries, limited resources and infrastructure often lead to incomplete registries, hindering the ability to gather consistent data and benchmark quality of care [74,75].

The European CF Society Patient Registry (ECFSPR) includes data from more than 54,000 CF patients, from 40 participating countries, reflecting the reality of CF across Europe [69]. Apart from the ECFSPR, many countries have their own CF national registry, improving transitional care [65,76,77,78,79].

Between 2011 and 2021, data collected by the ECFSPR from 27 European countries revealed significant trends in CF care and outcomes. These countries, stratified into lower-, middle-, and higher-income groups, showed a 60% increase in adult people with CF. The patient age distribution shifted toward older ages, indicating improved longevity even before novel therapies became available. Data analysis confirmed a significant decrease in mortality risk during 2019–2021, with hazard ratios consistently below one. However, the growth in adult CF patients and survival improvements were predominantly seen in higher- and middle-income countries, alongside better FEV1 % predicted values, underscoring disparities in outcomes across income groups [80].

In Romania, while CF has an estimated incidence of 1 in 2056 births, there is a lack of recent data on CF survival, limiting insights into patient outcomes and trends. This gap highlights the need for improved data collection and reporting to better understand and address the challenges faced by the CF community in Romania [81].

Notably, low-income countries, like Macedonia, Moldova, and Romania, as well as middle-income countries, like Latvia, Slovakia, and Slovenia, do not report any deaths among CF patients during childhood or adolescence [82].

Enhancing CF registries across all European countries could significantly improve the transition process for young adults with CF, promoting smoother, well-coordinated transitions and facilitating better long-term outcomes.

### 3.3. Australia and New Zealand

Australia and New Zealand launched a transition policy calling on primary, secondary, and tertiary healthcare providers to integrate coordinated, youth-centered community and school-based, primary, and specialty health services [83,84].

The Royal Australasian College of Physicians’ recommendations on transition emphasize that young people with chronic illnesses or disabilities should have a designated healthcare provider responsible for coordinating their shift to adult care. This includes creating detailed, individualized transition plans with input from both families and adolescents, ensuring seamless integration of community, primary, specialty, and allied health services. Adult care providers should assume responsibility for ongoing case management and follow-up, aided by accessible medical summaries to ensure continuity between healthcare professionals. Comprehensive, affordable healthcare must be accessible into adulthood, and young people with chronic conditions should receive the same standards of preventive care as their healthy peers, including holistic wellness assessments. Confidentiality is crucial during transitions, requiring thoughtful adherence to privacy and consent [83].

The Australian CF Data Registry reports the successful introduction of telemedicine in the management of CF patients, improving patients’ quality of life by reducing travel necessity and interruptions from work or education. Furthermore, telehealth services provide easier access to cross-disciplinary teams, particularly for patients living at significant distances from a tertiary CF clinic as it seems that, in Australia, 30% of people live outside of cities [85,86].

The Standards of Care for CF published in 2023 with the endorsement of the Thoracic Society of Australia and New Zealand launch special guidelines on the transition of CF patients. According to these recommendations, the process should start at the moment of diagnosis by introducing the parents to the concept of long-term management and transition, and it should include the patients around the age of 12–13. Starting with the age of 13–14, the adolescent should be gradually involved in individual consultations. The same authors recommend including a transition coordinator, organizing visits with the adult team including orientation tours to the adult facilities, exposing the adolescents to written and verbal information on the disease, the importance of treatment adherence, and possible complications. The transfer is complete after the first appointment with the adult team, and the patient is officially switched from the pediatric to the adult-oriented healthcare team [87].

Several pediatric centers and hospitals, such as the Children’s Hospital at Westmead in Sydney, adopted and successfully implemented the recommendations on transition by creating a unit-based protocol starting at the age of 12, with active transition at the age of 16 [10].

Results from a study which analyzed the medical records from all CF patients who transitioned from the Princess Margaret Hospital for Children to Sir Charles Gairdner Hospital (the adult CF center) between 2008 and 2012 revealed that the mean age at the moment of the transfer was 18.9 years, ranging between 17 and 22. The results show that transferring patients through a structured and individualized transition process promotes disease stability and quality of life [88].

### 3.4. Asia

In Asia, CF shows significant genetic diversity, with variations in the CFTR gene mutation and CF incidence differing by region and ethnicity. Contributing factors to this variability include under-reporting of cases, the absence of national registries, and differences in the carrier rates of CF mutations across countries [89,90].

In the East Asian population, CF remains relatively rare due to lower prevalence, with many cases often being undiagnosed [91,92]. China does not currently have a network of specialized CF centers, but there is a growing interest in translational practices in chronically ill teenagers, with general recommendations being made available [93]. The Chinese pediatric healthcare services typically provide care up to the age of 18, while adult healthcare systems begin at the age of 14, forming a gap between the ages of 14 and 18, where individuals must choose between the two available medical facilities. This choice is, however, left to the preference of the patients and their families, without a systematic referral support from the government or active healthcare associations [94,95].

A similar situation can be found in Japan, where CF is very rare and extensive CF centers are not available. Instead, treatment is typically provided within general hospitals by pulmonologists or gastroenterologists. The country has made strides in improving diagnosis, with genetic testing playing a crucial role in identifying rare CF mutations unique to Japanese patients [13,96].

Older data show that 62.3% of Japanese, chronically ill young adults aged 20 and older did not have a stable medical center and regularly addressed to pediatricians for the management of their chronic disease, indicating important dysfunctionalities in transitional care [97,98].

In 2014, the Japan Pediatric Society published the recommendations on transitional care for patients with childhood-onset chronic diseases, raising the interest for transitional care among healthcare specialists [99]. However, according to data from 2022, specialized protocols in alignment with international recommendations have been implemented in only five hospitals by the government’s Transition Support Model Project [98,100]. In 2019, additional transition guidelines have been published and distributed to healthcare facilities [101,102].

Similar data are available from Turkey as well, where transition policies are unavailable, with only preliminary data from isolated centers being available [103]. Researchers implemented a transition program in the Marmara CF center based on the American CFRISE model. During the process, a transition team was formed, consisting of pediatric pulmonologists, CF nurses, dietitians, physiotherapists, and one patient from the Turkish CF Association [39]. Preliminary data showed the successful implementation of the program, with the authors aiming at extending the project to include patients aged between 16 and 25 who received face-to-face transitional education every three months [104]. Other Turkish authors correlated the transition readiness assessment questionnaire scores with self-efficacy scales in CF patients aged between 14 and 17, proving a positive correlation between readiness scores and self-efficacy levels [105]. Such pioneering projects are supported by the implementation of the CF Registry of Turkey by the Turkish Pediatric Respiratory Diseases and CF Society in 2007 [106,107].

In India, although the interest for healthcare transition existed, active discussion and studies on the topic have only recently started [108,109,110,111,112]. In March 2024, the Indian Academy of Pediatrics in collaboration with the Directorate General of Health Services and the Government of India released an authoritative report on the healthcare transition of young patients with special healthcare needs, including chronic disease in adolescents [113].

### 3.5. Latin America

In Latin America, CF is two to three times less frequent than in the US or Europe [114]. Historically, CF has been perceived as rare in Latin America, potentially contributing to low detection rates due to a diminished index of suspicion, a misconception that requires urgent correction. Under-diagnosis of CF remains a significant challenge in Latin America, despite notable advancements in recent years [115,116].

While progress has been made, diagnostic practices across Latin America remain inconsistent. Neonatal screening programs are not universally implemented, and their establishment faces considerable logistical and systemic challenges. Expanding access to neonatal screening will require the development and enforcement of targeted health policies. Additionally, access to state-of-the-art diagnostic tools varies both between and within countries [117,118].

The genetic landscape of CF in Latin America is notably complex. Each country exhibits a diverse spectrum of CFTR mutations, reflecting both unique, country-specific variants and those linked to the population’s ethnic origins. Mexico, in particular, is notable for possessing one of the broadest known ranges of CFTR mutations globally. This underscores the need for broader disease awareness and improved understanding across the region [119].

Here, only a few countries have protocols or emerging models for transitioning CF patients, although these are generally in the early stages compared to those in North America or Europe. The most important CF centers are in Brazil, where multidisciplinary teams are in charge of gradually preparing adolescents for the transition to adult healthcare [120].

Researchers emphasize the fact that it is essential to extend pediatric care beyond the age of 18 and establish a legal framework allowing adolescents with chronic conditions to receive pediatric services up to the age of 25. Numerous countries have introduced health policies to support continuous developmental care and prevent segmenting care into stages, particularly during critical growth periods such as adolescence. For instance, Chile’s Ministry of Health allows pediatric services throughout the healthcare network until 19 years, 11 months, and 29 days (as per Order C/21 No. 1791 from 14 June 2012) [121].

Among the barriers encountered in CF care, countries from Latin America face difficulties related to treatment accessibility. For example, novel, promising therapies were approved in Brazil only in March 2022, after multiple petitions and pressure from organizations advocating for people living with CF such as the United for Life Institute [122].

**Figure 1:**
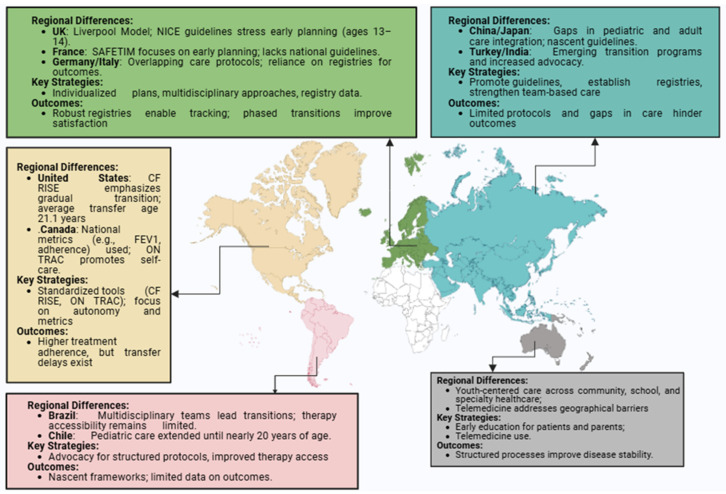
Global overview of CF transition care.

A panoramic representation of CF transitional practice is illustrated in Figure 1.

## 4. Discussion

Pediatric and adult CF teams should establish shared goals and guidelines to support a cohesive transition process for young patients. Collaborative efforts, with shared responsibility across both teams, are key to facilitating effective transitions. While defining a specific transition age is challenging due to varying practices across health systems, transition preparation should begin independently of age. Regular meetings between pediatric and adult teams to discuss upcoming transitions are ideal, though logistical constraints may occasionally limit in-person coordination.

Contraception is an important topic that should be addressed during counseling sessions with adolescents to provide personalized guidance, especially given thromboembolic risks. This is critical for those with comorbidities such as CF-related diabetes, osteoporosis, hepato-biliary disease, liver dysfunction, and pulmonary hypertension. Data show that, with consistent adherence to pancreatic enzyme replacement therapy, oral contraceptive pill malabsorption does not appear to pose a significant concern in CF patients [10].

Additionally, adolescents living with CF should be advised to take additional precautions against sexually transmitted infections, such as Chlamydia, Human Papillomavirus, hepatitis B, C, and HIV, as these could affect future eligibility for lung transplantation. Human Papillomavirus infection can lead to immunosuppression, carrying substantial long-term health risks, so maintaining up-to-date vaccinations is essential, including catch-up vaccinations when necessary to ensure comprehensive protection [10,123].

Reproductive medicine represents another topic of discussion during transition. Subfertility affects approximately 35% of women with CF, compared to rates of 5–15% in the general population. Given the high risk of obstetric complications, women with CF often require more intensive treatment and frequent outpatient visits throughout their pregnancy [124]. Realistic discussions regarding the current maternal health and fetal outcomes are of paramount importance. Counseling should not be limited to the patients, and the potential future fathers should also be included as genetic testing remains mandatory for both the patient and their partners, preferably using next generation sequencing methods [125,126].

In the CF population, numerous clinical factors have been investigated for their influence on health-related quality of life. Variables such as lung function, gender, body mass index, age, and the frequency of pulmonary exacerbations have been shown to correlate significantly with the different domains of health-related quality of life, reflecting the intricate interplay between clinical status and quality of life outcomes [127].

An epidemiological analysis of CF individuals identified significantly elevated rates of depression and anxiety, occurring at frequencies two to three times higher than those observed in the general population [128].

Furthermore, untreated depression and anxiety in people with CF have been associated with negative impacts on health-related quality of life, reduced adherence to treatment protocols, poorer medical outcomes, and increased healthcare expenditures. Consequently, international mental health guidelines, established by the CF Foundation and the European CF Society, advocate for the routine screening, management, and prevention of depression and anxiety as integral components of standard CF care [129,130].

While initiation of novel therapies has been associated with improved quality of life, many patients reported experiencing a sense of identity loss and the need to “redefine” their sense of self. This adjustment may feel misaligned with the optimistic expectations of family members and healthcare professionals. Additionally, related behavioral changes, such as modifications to dietary habits and increased engagement with academic or occupational responsibilities, can present significant challenges. These findings underscore the importance of addressing the psychosocial adjustment to this new health status with sensitivity and individualized support [131,132].

Such integrative approach to the transition process leads to the improvement of treatment adherence. During adolescence, the rates of treatment adherence in CF patients vary between 22% and 71%, depending on the increasing treatment complexity, socio-economic difficulties, limited resources and limited understanding of the role of follow-up medications, denial of the chronic disease, and poor mental health [133].

In middle- to low-income countries, the economic challenges in providing transitional CF care are significant. Limited healthcare funding often prioritizes basic CF management over specialized, age-specific transitional programs, leaving adolescent and adult CF patients with insufficient support. The high costs of CF treatments, compounded by scarce resources for multidisciplinary teams, hinder consistent, long-term care. Many healthcare systems in these regions lack the infrastructure and financial flexibility to support the development of dedicated adult CF care units, further complicating continuity in patient management as young patients age [134,135,136].

Governmental support towards transition policies represents the key to funding allocation and program implementation. In December 2021, the United Nations adopted the first resolution for chronic patients living with rare diseases, ‘Addressing the Challenges of Persons Living with a Rare Disease and their Families’. This resolution, which was unanimously adopted by all 193 United Nations’ members, represents a milestone for coordinated and robust patient care. Such initiatives encourage governments to adapt national healthcare policies, including structured, patient-centered transitional programs, bridging the gaps for continuous medical care [122,137].

International CF transition models share a common goal of ensuring a smooth and effective transition from pediatric to adult care. Different models often base their recommendations on specific developmental, clinical, and psychosocial criteria. For instance, many emphasize the importance of achieving developmental readiness, which includes understanding one’s own health condition, mastering self-management skills, and demonstrating autonomy in decision-making [29,138,139].

Despite variations in healthcare systems and cultural contexts, international CF transition models often agree on certain benchmarks. These include the need for early preparation, typically beginning in early adolescence, and the importance of a structured, multidisciplinary approach involving both pediatric and adult care teams. Transition readiness assessments, often conducted through standardized tools or questionnaires, are universally recommended to identify gaps in self-management skills or knowledge. Furthermore, clear communication and collaboration between pediatric and adult providers are consistently emphasized as critical for the continuity of care. Regular follow-ups during the transition phase and a designated transition coordinator are additional elements that many models advocate to facilitate the process and minimize the risk of care disruption.

## 5. Conclusions

The transition from pediatric to adult care in CF is a complex but essential process that requires a structured, patient-centered approach. By examining transition models across different regions, this article highlights key strategies and challenges that impact successful outcomes, such as early preparation, interdisciplinary coordination, and family involvement. Despite regional variations, patient education, the gradual transfer of responsibility, and the continuity of care are essential for successful transitions.

Effective transitions in CF patients hold immense potential for improving long-term outcomes while simultaneously reducing healthcare costs. Well-coordinated transitions mitigate the risks of treatment interruptions, lapses in medication adherence, and preventable complications. By bridging transitional gaps, healthcare systems can ensure sustainable care delivery, improve quality of life for individuals with CF, and achieve cost savings through the prevention of disease-related complications.

To address global disparities, collaboration among stakeholders is essential to develop standardized, scalable transition models tailored to regional needs, ultimately improving quality of life and long-term health outcomes for individuals with CF.

## Data Availability

Not applicable.
